# Supplementary material for: Loss-of-Function Variants in DRD1 in Infantile Parkinsonism-Dystonia
Source: Cells. 2023 Mar 30;12(7):1046. doi: 10.3390/cells12071046 (PMC10093404; doi:10.3390/cells12071046)
Supplement: Supplementary file 1 [file cells-12-01046-s001.zip › cells-2218643-supplementary.pdf]

Loss-of-Function Variants in *DRD1* in Infantile Parkinsonism-Dystonia

Kimberley M. Reid [1,†], Dora Steel [1,2,†], Sanjana Nair [3], Sanjay Bhate [2,4], Lorenzo Biassoni [2,4], Sniya Sudhakar [2,4], Michelle Heys [5,6], Elizabeth Burke [7], Erik-Jan Kamsteeg [8], Genomics England Research Consortium [9,10], Biju Hameed [2], Michael Zech [11,12,] Niccolo E. Mencacci [13], Katy Barwick [1], Maya Topf [3] and Manju A. Kurian [1,2,*]

[1] Molecular Neurosciences, Developmental Neurosciences, Zayed Centre for Research into Rare Disease in Children, UCL GOS Institute of Child Health, London WC1N 1DZ, UK
[2] Department of Neurology, Great Ormond Street Hospital, London WC1N 3JH, UK
[3] Leibniz Institute of Virology (LIV) and Universitätsklinikum Hamburg Eppendorf (UKE), Centre for Structural Systems Biology (CSSB), 20251 Hamburg, Germany
[4] Department of Radiology, Great Ormond Street Hospital, London WC1N 3JH, UK
[5] Department of Population, Policy and Practice, UCL GOS Institute of Child Health, London WC1N 1DZ, UK
[6] Specialist Children's and Young People's Services, Newham, East London NHS Foundation Trust, London RM13 8GQ, UK
[7] Office of the Clinical Director, National Human Genome Research Institute, Undiagnosed Diseases Program and Network, Office of the Director, National Institutes of Health, Bethesda, MD 20892, USA
[8] Department of Human Genetics, Radboud University Medical Center, 6525 Nijmegen, The Netherlands
[9] Genomics England, London EC1M 6BQ, UK
[10] William Harvey Research Institute, Queen Mary University of London, London EC1M 6BQ, UK
[11] Institute of Human Genetics, School of Medicine, Technical University of Munich, 85354 Munich, Germany
[12] Institute of Neurogenomics, Helmholtz Zentrum München, 85764 Munich, Germany
[13] Feinberg School of Medicine, Northwestern University, Chicago IL 60611, USA
† These authors contributed equally to this work.

Supplementary Information

Supplementary tables……………………………………………….…………………3
Supplementary figures and legends……………………………….…………..11

SUPPLEMENTARY TABLES

Table S1: Genes causing neurodevelopmental syndromes, movement disorders, and epilepsy phenotypes, interrogated on whole-genome sequencing

A2ML1	ADAR1	ALDH4A1	AP4E1	ASMT	ATP6V0A1	B4GALT7	BSND	CAP1	CDKN1C	CHUK	COG8
AAAS	ADAT3	ALDH5A1	AP4M1	ASMTL	ATP6V0A2	B4GAT1	BTD	CAPN1	CDON	CIB2	COL10A1
AAK1	ADCK3	ALDH6A1	AP4S1	ASNS	ATP6V0A4	B9D1	BTK	CAPN10	CDT1	CIC	COL11A1
AARS	ADCK4	ALDH7A1	AP5Z1	ASPA	ATP6V0B	B9D1	BUB1B	CAPRIN1	CEND1	CISD2	COL11A2
AARS2	ADCY1	ALDOA	APOA1	ASPH	ATP6V0C	B9D2	C10orf2	CAPZA2	CENPF	CIT	COL18A1
AASS	ADCY2	ALDOB	APOA5	ASPM	ATP6V0D1	BAAT	C12orf4	CAPZB	CENPJ	CIZ1	COL1A1
ABAT	ADCY3	ALG1	APOB	ASS1	ATP6V0D2	BAG3	C12orf57	CARS2	CEP104	CKAP2L	COL1A2
ABCA1	ADCY4	ALG11	APOC2	ASXL1	ATP6V0E1	BBIP1	C12orf65	CASK	CEP120	CKB	COL25A1
ABCB11	ADCY5	ALG12	APOE	ASXL2	ATP6V0E2	BBS1	C19orf12	CASK	CEP135	CLCN1	COL2A1
ABCB4	ADCY6	ALG13	APOPT1	ASXL3	ATP6V1A	BBS10	C19orf12	CASP2	CEP152	CLCN2	COL4A1
ABCB7	ADCY7	ALG14	APP	ATAD3A	ATP6V1B1	BBS12	C19orf70	CASQ2	CEP164	CLCN3	COL4A2
ABCC6	ADCY8	ALG2	APRT	ATAD3B	ATP6V1B2	BBS2	C1GALT1C1	CASR	CEP290	CLCN4	COL4A3
ABCC8	ADCY9	ALG3	APTX	ATCAY	ATP6V1C1	BBS4	C1QBP	CAT	CEP41	CLCN5	COL4A3BP
ABCC9	ADGRG1	ALG6	AQP7	ATF2	ATP6V1C2	BBS5	C21orf2	CAV3	CEP57	CLCN6	COL4A4
ABCD1	ADGRG4	ALG8	AR	ATF4	ATP6V1D	BBS7	C21orf59	CBL	CEP63	CLCN7	COL4A6
ABCD4	ADGRG6	ALG9	ARAF	ATF6B	ATP6V1E1	BBS9	C2CD3	CBS	CEP83	CLCNKA	COL6A1
ABCG2	ADGRV1	ALMS1	ARCN1	ATG2A	ATP6V1E2	BCAP31	C2orf71	CC2D1A	CEP89	CLCNKB	COL6A3
ABCG5	ADK	ALOX12	ARFGAP1	ATG2B	ATP6V1F	BCAT1	C3orf58	CC2D2A	CERS1	CLDN16	COL9A1
ABCG8	ADNP	ALOX12B	ARFGEF2	ATIC	ATP6V1G1	BCAT2	C4BPA	CCBE1	CETP	CLDN19	COL9A2
ABHD12	ADPRHL2	ALOX15	ARFGEF2	ATL1	ATP6V1G2	BCKDHA	C4orf26	CCDC103	CFAP43	CLIC2	COL9A3
ABHD5	ADRA2B	ALOX15B	ARFIP2	ATL3	ATP6V1G3	BCKDHB	C5orf42	CCDC114	CFAP44	CLN3	COLEC10
ACAD8	ADSL	ALOX5	ARG1	ATM	ATP6V1H	BCKDK	C6orf136	CCDC115	CFAP47	CLN5	COLEC11
ACAD9	AFF2	ALPL	ARHGAP1	ATN1	ATP6VA4	BCL11A	C8orf37	CCDC151	CFAP53	CLN6	COMP
ACADM	AFF3	ALS2	ARHGAP31	ATP10A	ATP7A	BCL11B	C9orf72	CCDC22	CFC1	CLN8	COMT
ACADS	AFF4	ALX1	ARHGAP36	ATP10B	ATP7B	BCL2	CA2	CCDC28B	CFP	CLOCK	COQ2
ACADSB	AFG3L2	ALX3	ARHGAP6	ATP10D	ATP7G	BCOR	CA5A	CCDC39	CFTR	CLP1	COQ4
ACADVL	AFP	ALX4	ARHGEF10	ATP11A	ATP8A1	BCORL1	CA8	CCDC40	CHAMP1	CLPB	COQ5
ACAN	AGA	AMACR	ARHGEF2	ATP11B	ATP8A2	BCS1L	CACNA1A	CCDC47	CHAT	CLPP	COQ6
ACAT1	AGK	AMER1	ARHGEF4	ATP11C	ATP8B1	BDNF	CACNA1B	CCDC65	CHCHD10	CLPS	COQ7
ACBD5	AGL	AMN	ARHGEF6	ATP13A2	ATP8B2	BDP1	CACNA1C	CCDC78	CHCHD2	CLRN1	COQ8A
ACBD6	AGO1	AMPD1	ARHGEF9	ATP1A1	ATP8B3	BEAN1	CACNA1D	CCDC8	CHD2	CLTC	COQ8B
ACE2	AGPAT2	AMPD2	ARID1A	ATP1A2	ATP8B4	BFSP2	CACNA1E	CCDC88A	CHD3	CLTCL1	COQ9
ACER2	AGPS	AMT	ARID1B	ATP1A3	ATP9A	BGN	CACNA1F	CCDC88C	CHD4	CMC4	COX10
ACHE	AGT	AN03	ARID2	ATP1B1	ATP9B	BHLHA9	CACNA1G	CCNA2	CHD7	CNBP	COX11
ACIN1	AGTR2	ANG	ARIH1	ATP2A1	ATPAF1	BICD2	CACNA1H	CCNB3	CHD8	CNDP1	COX14
ACO2	AGXT	ANK2	ARL10	ATP2A2	ATPAF2	BIN1	CACNA1S	CCND2	CHKB	CNKSR1	COX15
ACOT9	AHCY	ANK3	ARL13B	ATP2B1	ATR	BLM	CACNA2D1	CCNO	CHL1	CNKSR2	COX16
ACOX1	AHDC1	ANKH	ARL14EP	ATP2B2	ATRX	BMP15	CACNA2D2	CCT5	CHM	CNNM2	COX17
ACP5	AHI1	ANKRD1	ARL6	ATP2B3	ATXN1	BMP4	CACNA2D3	CD320	CHMP1A	CNOT3	COX18
ACSF3	AIFM1	ANKRD11	ARL6IP1	ATP2C1	ATXN10	BMPER	CACNB3	CD47	CHMP2B	CNPY3	COX19
ACSL4	AIMP1	ANKS6	ARL8A	ATP2C2	ATXN2	BMPR1B	CACNB4	CD96	CHP1	CNTN1	COX20
ACTA1	AIMP2	ANO10	ARMC4	ATP5A1	ATXN3	BOLA1	CACNG2	CD99	CHRDL1	CNTN2	COX4I1
ACTA2	AIPL1	ANO3	ARMC9	ATP5B	ATXN3L	BOLA2	CAD	CDC42	CHRM1	CNTN2	COX4I2
ACTB	AIRE	ANO5	ARNTL	ATP5C1	ATXN7	BOLA3	CADM3	CDC45	CHRM2	CNTN3	COX5A
ACTB	AK1	ANTXR1	ARRB2	ATP5D	ATXN8	BORCS5	CALM1	CDC6	CHRM3	CNTN4	COX5B
ACTC1	AK2	AOX1	ARSA	ATP5E	AUH	BPIFB6	CALM2	CDH11	CHRM4	CNTNAP1	COX6A1
ACTG1	AKAP17A	AP1B1	ARSB	ATP5F1	AUTS2	BPTF	CALM3	CDH13	CHRM5	CNTNAP2	COX6A2
ACTL6A	AKAP4	AP1S1	ARSE	ATP5F1A	AVP	BRAF	CALML3	CDH15	CHRNA2	COA1	COX6B1
ACTL6B	AKAP6	AP1S2	ARSF	ATP5G1	AVPR1A	BRAT1	CALML4	CDH23	CHRNA3	COA3	COX6B2
ACTN1	AKR1C2	AP2A1	ARSG	ATP5G2	AVPR1B	BRCA1	CALML5	CDH3	CHRNA4	COA4	COX6C
ACTN2	AKR1D1	AP2A2	ARSI	ATP5G3	AVPR2	BRCA2	CALML6	CDK10	CHRNA6	COA5	COX7A1
ACTR1A	AKT1	AP2B1	ARV1	ATP5H	AWAT2	BRDT	CALY	CDK13	CHRNA7	COA6	COX7A2
ACTR2	AKT2	AP2M1	ARX	ATP5I	B3GALNT2	BRF1	CAMK2A	CDK16	CHRNB1	COA7	COX7B
ACTR3	AKT3	AP2S1	ARX	ATP5J	B3GALT6	BRIP1	CAMK2B	CDK19	CHRNB2	COASY	COX7B2
ACTR5	ALAD	AP3B1	ASAH1	ATP5J2	B3GALTL	BRPF1	CAMK2D	CDK5	CHRNB4	COG1	COX7C
ACVR1	ALAS2	AP3B2	ASB12	ATP5L	B3GAT3	BRPF3	CAMK2G	CDK5R1	CHRNG	COG2	COX8A
ACVR2B	ALDH18A1	AP3D1	ASCC3	ATP5L2	B3GLCT	BRWD3	CAMK4	CDK5RAP2	CHST14	COG4	CP
ACY1	ALDH1A3	AP3M2	ASCL1	ATP5O	B3GNT1	BSCL2	CAMTA1	CDK5RAP2	CHST3	COG5	CPA6
ADA	ALDH1B1	AP3S2	ASH1L	ATP6AP1	B4GALNT1	BSG	CANT1	CDK8	CHST6	COG6	CPD
ADAR	ALDH3A2	AP4B1	ASL	ATP6AP2	B4GALT1	BSN	CANX	CDKL5	CHSY1	COG7	CPLX1

CPLX2	CTSF	DENND5A	DNAJC5	ECEL1	ERF	FBP2	FREM1	GAN	GLS	GRIA1	HAX1
CPLX3	CTSK	DEPDC5	DNAJC6	ECHS1	ERLIN1	FBXL4	FREM2	GAP43	GLS2	GRIA2	HCCS
CPLX4	CTTNBP2	DES	DNAJC6	ECSIT	ERLIN2	FBXO11	FRMD4A	GAPDH	GLUD1	GRIA3	HCFC1
CPOX	CUBN	DGKH	DNAL1	EDA	ERMARD	FBXO25	FRMD7	GARS	GLUL	GRIA4	HCN1
CPS1	CUL3	DGUOK	DNHD1	EDNRA	ERN1	FBXO38	FRMPD4	GAS8	GLYCTK	GRID2	HCN2
CPT1A	CUL4B	DHCR24	DNM1	EDNRB	ESCO2	FBXO7	FRRS1L	GATA2	GM2A	GRIK1	HCRT
CPT2	CUL7	DHCR7	DNM1L	EED	ESX1	FBXO8	FRY	GATA4	GMPPA	GRIK2	HDAC4
CPXCR1	CUX2	DHDDS	DNM2	EEF1A2	ETFA	FBXW4	FTCD	GATA6	GMPPB	GRIK3	HDAC6
CRADD	CWC27	DHFR	DNM3	EEF1B2	ETFB	FDX1L	FTL	GATAD1	GNA01	GRIK4	HDAC8
CRB1	CWF19L1	DHFR2	DNMT1	EFHC1	ETFDH	FDX2	FTO	GATAD2B	GNA11	GRIK5	HECW2
CRB2	CXorf58	DHFRL1	DNMT3A	EFNB1	ETHE1	FDXR	FTSJ1	GATB	GNA13	GRIN1	HEPACAM
CRBN	CYB5R3	DHH	DNMT3B	EFTUD2	EVC	FECH	FUCA1	GATC	GNAI1	GRIN2A	HERC1
CREB1	CYC1	DHODH	DOC2B	EGF	EVC2	FGD1	FUS	GATM	GNAI2	GRIN2B	HERC2
CREB3	CYCS	DHRSX	DOCK11	EGR2	EXOC3	FGD4	FUT8	GBA	GNAI3	GRIN2C	HESX1
CREB3L1	CYFIP2	DHTKD1	DOCK3	EHMT1	EXOC3L2	FGF10	FXN	GBA2	GNAL	GRIN2D	HEXA
CREB3L2	CYP1B1	DHX30	DOCK6	EIF2AK2	EXOC4	FGF12	FXYD2	GBE1	GNAO1	GRIN3A	HEXB
CREB3L3	CYP27A1	DIAPH1	DOCK7	EIF2AK3	EXOC8	FGF14	FYCO1	GCDH	GNAQ	GRIN3B	HFE
CREB3L4	CYP2C18	DIAPH2	DOCK8	EIF2B1	EXOSC3	FGF3	FYN	GCH1	GNAS	GRIP1	HFE2
CREB5	CYP2C19	DIP2B	DOLK	EIF2B2	EXOSC8	FGFR1	FZD3	GCH1	GNAZ	GRK2	HGD
CREBBP	CYP2C8	DIRAS2	DPAGT1	EIF2B3	EXOSC9	FGFR2	FZD6	GCK	GNB1	GRK3	HGSNAT
CRELD1	CYP2C9	DIS3L2	DPEP1	EIF2B4	EXT1	FGFR3	G14459A	GCLC	GNB2	GRM1	HIBCH
CRH	CYP2J2	DKC1	DPF1	EIF2B5	EXT2	FH	G6PC	GCN1L1	GNB3	GRM2	HINT1
CRLF2	CYP2U1	DLAT	DPF2	EIF2S3	EXTL3	FHL1	G6PC3	GCSH	GNB4	GRM3	HIST1H1E
CRX	CYP2UI	DLD	DPF3	EIF3F	EYA1	FIBP	GAA	GDAP1	GNB5	GRM4	HIST1H4B
CRYAA	CYP4X1	DLG1	DPM1	EIF3G	EZH2	FIG4	GAB3	GDAP2	GNE	GRM5	HIST1H4C
CRYAB	CYP7A1	DLG2	DPM2	EIF4A3	F5	FKBP14	GABARAP	GDF1	GNG10	GRM6	HIST3H3
CRYBA1	CYP7B1	DLG3	DPM3	EIF4G1	FA2H	FKBP6	GABARAPL1	GDF5	GNG11	GRM7	HIVEP2
CRYBA4	D2HGDH	DLG4	DPP6	ELAC2	FAAH2	FKBPL	GABARAPL2	GDF6	GNG12	GRM8	HK1
CRYBB1	DAB1	DLGAP1	DPYD	ELK1	FAAHP1	FKRP	GABBR1	GDI1	GNG13	GRN	HLA-DQB1
CRYBB2	DACT1	DLGAP2	DPYS	ELN	FAH	FKTN	GABBR2	GEMIN4	GNG2	GSK3A	HLCS
CRYBB3	DAG1	DLL3	DRC1	ELOVL4	FAHD2A	FLAD1	GABRA1	GFAP	GNG3	GSK3B	HMBS
CRYGC	DARS	DLL4	DRD1	ELOVL5	FAM111A	FLNA	GABRA2	GFER	GNG4	GSPT2	HMCN1
CRYGD	DARS2	DLST	DRD2	ELP1	FAM111B	FLNA	GABRA3	GFM1	GNG5	GSS	HMGB3
CSF1R	DBH	DMBX1	DRD3	ELP2	FAM120C	FLNB	GABRA4	GFM2	GNG7	GTF2E2	HMGCL
CSF1R	DBT	DMD	DRD4	EMD	FAM126A	FLOT1	GABRA5	GFPT1	GNG8	GTF2H5	HMGCS2
CSF2RA	DCAF10	DMGDH	DRD5	EML1	FAM161A	FLOT2	GABRA6	GGT1	GNGT1	GTF3C3	HNF1B
CSNK1D	DCAF17	DMP1	DRP2	EMX2	FAM193A	FLT4	GABRB1	GHR	GNGT2	GTF3C4	HNF4A
CSNK1E	DCAF8	DMPK	DSC2	EN2	FAM20A	FLVCR1	GABRB2	GIF	GNMT	GTPBP2	HNRNPA1
CSNK1G1	DCC	DMXL2	DSCAM	ENO3	FAM20C	FLVCR2	GABRB3	GIGYF2	GNPAT	GTPBP3	HNRNPH2
CSNK2A1	DCDC2	DNA2	DSG2	ENOX2	FAM213A	FMN2	GABRD	GJA1	GNPTAB	GTPBP8	HNRNPK
CSNK2B	DCHS1	DNAAF1	DSP	ENPP1	FAM47B	FMO3	GABRE	GJA3	GNPTG	GUCY2C	HNRNPR
CSPP1	DCHS2	DNAAF2	DSPP	ENTPD1	FAM58A	FMR1	GABRG1	GJA8	GNS	GUCY2D	HNRNPU
CSRP3	DCTN1	DNAAF3	DST	EOGT	FANCA	FOLR1	GABRG2	GJB1	GON4L	GUSB	HOGA1
CSTB	DCTN1	DNAAF4	DSTYK	EOMES	FANCB	FOLR2	GABRG3	GJB2	GORAB	GYG1	HOMER1
CSTF2	DCX	DNAAF5	DTD1	EP300	FANCC	FOLR3	GABRP	GJB3	GOSR2	GYS1	HOMER2
CTC1	DCXR	DNAH1	DTNA	EPB41L1	FANCD2	FOS	GABRQ	GJC2	GPAA1	GYS2	HOMER3
CTCF	DDB1	DNAH10	DUSP1	EPG5	FANCE	FOXC1	GABRR1	GK	GPC3	H3F3A	HOXA1
CTDP1	DDB2	DNAH11	DVL1	EPM2A	FANCF	FOXC2	GABRR2	GLA	GPD1	H3F3B	HOXA13
CTGF	DDC	DNAH5	DVL3	EPPK1	FANCG	FOXE1	GABRR3	GLB1	GPHN	HAAO	HOXC13
CTH	DDHD1	DNAH6	DYM	ERAL1	FANCI	FOXE3	GAD1	GLDC	GPI	HACE1	HOXD10
CTNNA1	DDHD2	DNAH9	DYNC1H1	ERBB4	FAR1	FOXF1	GAD2	GLE1	GPM6A	HADH	HOXD13
CTNNA2	DDOST	DNAI1	DYNC2H1	ERC2	FARS2	FOXG1	GAL	GLI2	GPM6B	HADHA	HPCA
CTNNB1	DDR2	DNAI2	DYNC2LI1	ERCC1	FASH	FOXH1	GALC	GLI3	GPR179	HADHB	HPCAL1
CTNND1	DDX11	DNAJA3	DYNLL1	ERCC2	FASN	FOXN1	GALE	GLIS2	GPR98	HAL	HPD
CTNND2	DDX3X	DNAJB13	DYRK1A	ERCC3	FASTKD2	FOXP1	GALK1	GLIS3	GPRASP1	HAMP	HPDL
CTNS	DDX53	DNAJB2	EARS2	ERCC4	FAT4	FOXP2	GALNS	GLMN	GPSM2	HAP1	HPGD
CTPS2	DDX58	DNAJC11	EBF1	ERCC5	FBLN5	FOXP3	GALNT12	GLRA1	GPSM2	HAPLN2	HPRT1
CTSA	DDX59	DNAJC12	EBF1	ERCC6	FBN1	FOXRED1	GALNT3	GLRA2	GRB14	HARS	HPS1
CTSC	DEAF1	DNAJC19	EBF3	ERCC6L2	FBN2	FR1	GALT	GLRB	GRHL3	HARS2	HPSE2
CTSD	DECR1	DNAJC3	EBP	ERCC8	FBP1	FRAS1	GAMT	GLRX5	GRHPR	HAUS7	HR

HRAS	IFT80	JMJD4	KCNK9	KMT2C	LMX1B	MAP6	MGAT5B	MT-ATP8	MYH14	NDUFAF5	NKX2-5
HS6ST2	IFT81	JMJD7	KCNMA1	KMT2D	LNPK	MAP7D3	MGME1	MT-CO1	MYH3	NDUFAF6	NKX3-2
HSD17B10	IGBP1	JPH2	KCNN2	KMT2E	LOH12CR1	MAPK1	MGP	MT-CO2	MYH6	NDUFAF7	NKX6-2
HSD17B4	IGF1	JPH3	KCNQ1	KMT5B	LONP1	MAPK10	MIB1	MT-CO3	MYH7	NDUFAF8	NLGN3
HSD3B7	IGF1R	JUP	KCNQ2	KNCJ6	LOXHD1	MAPK11	MICU1	MT-CYB	MYH8	NDUFB1	NLGN4X
HSF4	IGF2	KALRN	KCNQ3	KNL1	LPIN1	MAPK12	MID1	MTF1	MYH9	NDUFB10	NLRP3
HSP90AA1	IGHMBP2	KANK1	KCNQ4	KPTN	LPL	MAPK13	MIPEP	MTFMT	MYL2	NDUFB11	NME8
HSPA8	IGSF1	KANSL1	KCNQ5	KRAS	LRAT	MAPK14	MIR17HG	MTHFD1	MYL3	NDUFB2	NMNAT1
HSPA9	IHH	KARS	KCNS1	KRIT1	LRGUK	MAPK2	MITF	MTHFR	MYO1D	NDUFB3	NNT
HSPB1	IKBKG	KAT5	KCNS2	KYNU	LRP1	MAPK8	MKKS	MTHFS	MYO1G	NDUFB4	NODAL
HSPB3	IL11RA	KAT6A	KCNS3	L1CAM	LRP2	MAPK8IP3	MKS1	MTM1	MYO5A	NDUFB5	NOG
HSPB8	IL1RAPL1	KAT6B	KCNT1	L2HGDH	LRP4	MAPK9	MLC1	MTMR1	MYO5B	NDUFB6	NONO
HSPD1	IL1RAPL2	KATNAL2	KCNV1	LACTB	LRP5	MAPKBP1	MLH1	MTMR14	MYO7A	NDUFB7	NOP56
HSPG2	IL3RA	KBTBD13	KCNV2	LAMA1	LRPPRC	MAPRE2	MLYCD	MTMR2	MYOZ2	NDUFB8	NOTCH2
HTR1A	ILF2	KCNA1	KCTD1	LAMA2	LRRC17	MAPT	MMAA	MTMR8	MYPN	NDUFB9	NOTCH3
HTR1B	IMPAD1	KCNA10	KCTD17	LAMA4	LRRC6	MARS	MMAB	MT-ND1	MYT1	NDUFC1	NPC1
HTR1D	IMPDH1	KCNA2	KCTD3	LAMB1	LRRK1	MARS2	MMACHC	MT-ND2	MYT1L	NDUFC2	NPC2
HTR1E	INA	KCNA3	KCTD7	LAMC3	LRRK2	MASP1	MMADHC	MT-ND3	NAA10	NDUFS1	NPHP1
HTR1F	INF2	KCNA4	KDM1A	LAMP1	LRRK2	MAST1	MME	MT-ND4	NAA15	NDUFS2	NPHP3
HTR2A	INO80	KCNA5	KDM3B	LAMP2	LRSAM1	MAT1A	MMP13	MT-ND4L	NACC1	NDUFS3	NPHP4
HTR2B	INPP4A	KCNA6	KDM5A	LAP3	LSAMP	MATN3	MMP21	MT-ND5	NADK2	NDUFS4	NPHS1
HTR2C	INPP5E	KCNA7	KDM5B	LARGE	LTBP2	MATN4	MNX1	MT-ND6	NAGA	NDUFS5	NPHS2
HTR3A	INPP5K	KCNB1	KDM5C	LARGE1	LTBP3	MBD5	MOCS1	MTO1	NAGLU	NDUFS6	NPR2
HTR3B	INPPL1	KCNB2	KDM6A	LARP7	LYRM4	MBL2	MOCS2	MTOR	NAGS	NDUFS7	NPR3
HTR3C	INSR	KCNC1	KDM6B	LARS	LYRM7	MBNL3	MOG	MTPAP	NALCN	NDUFS8	NPRL2
HTR3D	INTS1	KCNC2	KHK	LARS2	LYST	MBOAT7	MOGS	MTR	NANS	NDUFV1	NPRL3
HTR3E	INTS6	KCNC3	KIAA0556	LAS1L	LZTFL1	MBTPS2	MORC2	MT-RNR1	NAPA	NDUFV2	NPTN
HTR4	INTS6L	KCNC4	KIAA0586	LBR	LZTR1	MC2R	MORC4	MT-RNR2	NAPB	NDUFV3	NR1I3
HTR5A	INTS8	KCND1	KIAA0753	LCA5	MAB21L1	MCCC1	MOSS2	MTRR	NARS2	NEB	NR2F1
HTR6	INVS	KCND2	KIAA1109	LCAT	MAB21L2	MCCC2	MPC1	MT-TA	NAT8L	NEBL	NR2F2
HTR7	IPPK	KCND3	KIAA1279	LCT	MACC1	MCEE	MPDU1	MT-TC	NAXE	NECAB2	NR4A2
HTRA1	IQCB1	KCNE1	KIDINS220	LDB3	MACF1	MCIDAS	MPDZ	MT-TD	NBAS	NECAP1	NR5A1
HTRA2	IQSEC2	KCNF1	KIF11	LDHA	MADD	MCM3AP	MPI	MT-TE	NBEA	NECTIN1	NRAS
HTT	IRAK1	KCNG1	KIF14	LDLR	MAF	MCM9	MPLKIP	MT-TF	NBN	NEDD4L	NRG2
HUWE1	IREB2	KCNG2	KIF1A	LDLRAP1	MAFB	MCOLN1	MPV17	MT-TG	NCAM1	NEFH	NRK
HYAL1	IRF2BPL	KCNG3	KIF1B	LEFTY2	MAG	MCPH1	MPZ	MT-TH	NCAM2	NEFL	NRXN1
HYDIN	IRF6	KCNG4	KIF1BP	LEMD3	MAGEA11	MDH2	MR1	MT-TI	NCKAP1	NEGR1	NRXN2
HYKK	IRX5	KCNH1	KIF1C	LETM1	MAGEB1	MECP2	MRAP	MT-TK	NDE1	NEK1	NRXN3
HYLS1	ISCA1	KCNH2	KIF21A	LFNG	MAGEB10	MECR	MRE11	MT-TL1	NDN	NEK8	NSD1
IARS	ISCA2	KCNH3	KIF22	LGI1	MAGEB2	MED12	MRE11A	MT-TL2	NDP	NEU1	NSD2
IARS2	ISCU	KCNH4	KIF26B	LGI4	MAGEC1	MED13L	MRM2	MT-TM	NDRG1	NEXMIF	NSDHL
IBA57	ISG15	KCNH5	KIF2A	LHFPL3	MAGEC3	MED17	MRPL12	MT-TN	NDST1	NEXN	NSF
ICAM5	ISPD	KCNH6	KIF4A	LHX3	MAGED1	MED23	MRPL3	MTTP	NDUFA1	NF1	NSUN2
ICK	ITCH	KCNH7	KIF5A	LHX4	MAGEE2	MED25	MRPL40	MT-TP	NDUFA10	NFASC	NSUN3
IDH2	ITGA3	KCNH8	KIF5B	LIAS	MAGEL2	MEF2C	MRPL44	MT-TQ	NDUFA11	NFIA	NT5C
IDH3A	ITGA4	KCNJ10	KIF5C	LIG4	MAGI2	MEG3	MRPS14	MT-TR	NDUFA12	NFIB	NT5C2
IDH3B	ITGA7	KCNJ11	KIF7	LIMK1	MAGIX	MEGF10	MRPS16	MT-TS1	NDUFA13	NFIX	NT5C3A
IDS	ITGB6	KCNJ12	KIRREL3	LINGO1	MAGT1	MEGF8	MRPS2	MT-TS2	NDUFA2	NFS1	NTM
IDUA	ITIH6	KCNJ13	KIT	LINS1	MAL2	MEIS2	MRPS22	MT-TT	NDUFA3	NFU1	NTNG1
IER3IP1	ITM2B	KCNJ14	KLC4	LIPA	MAN1B1	MEOX2	MRPS23	MT-TV	NDUFA4	NGF	NTRK1
IFIH1	ITPA	KCNJ18	KLF1	LIPC	MAN2B1	MESP2	MRPS34	MT-TW	NDUFA5	NGLY1	NTRK2
IFITM5	ITPR1	KCNJ2	KLF8	LIPI	MANBA	MET	MRPS7	MT-TY	NDUFA6	NHEJ1	NUBPL
IFNAR2	ITPR2	KCNJ3	KLHL15	LIPT1	MAOA	METTL23	MSFD8	MUC1	NDUFA7	NHLRC1	NUDT2
IFT122	ITPR3	KCNJ4	KLHL21	LIPT2	MAOB	MFF	MSL3	MUT	NDUFA8	NHP2	NUP107
IFT140	IVD	KCNJ5	KLHL34	LITAF	MAP1A	MFN2	MSMO1	MVK	NDUFA9	NHS	NUP188
IFT172	JAG1	KCNJ6	KLHL4	LMAN1	MAP1B	MFRP	MSTO1	MXRA5	NDUFAB1	NID1	NUP62
IFT27	JAGN1	KCNJ9	KLHL40	LMBRD1	MAP2K1	MFSD2A	MSX1	MYBPC1	NDUFAF1	NIPA1	NUS1
IFT43	JAK2	KCNK12	KLHL7	LMNA	MAP2K2	MFSD4B	MSX2	MYBPC3	NDUFAF2	NIPBL	NXF4
IFT52	JAK3	KCNK18	KMT2A	LMNB1	MAP3K1	MFSD8	MTATP6	MYCN	NDUFAF3	NKAP	NXF5
IFT74	JAM3	KCNK4	KMT2B	LMNB2	MAP3K15	MGAT2	MT-ATP6	MYH10	NDUFAF4	NKX2-1	NYX

OAT	PCBD1	PFKM	PJA1	POLR1A	PRKACG	PYGM	RAPGEF1	RORA	SCN1B	SHPK	SLC25A46
OBSL1	PCCA	PFN1	PKAN	POLR1C	PRKAG2	QARS	RAPGEF3	RORB	SCN2A	SHROOM2	SLC25A53
OCIAD1	PCCB	PGAM2	PKD1	POLR1D	PRKAR1A	QDPR	RAPSN	RP1	SCN2B	SHROOM4	SLC25A6
OCLN	PCDH10	PGAP1	PKD1L1	POLR3A	PRKCA	QKI	RARB	RPE65	SCN3A	SI	SLC26A2
OCRL	PCDH12	PGAP2	PKD2	POLR3B	PRKCB	QRICH1	RARS	RPGR	SCN4A	SIGMAR1	SLC26A9
ODC1	PCDH15	PGAP3	PKHD1	POMGNT1	PRKCE	QRSL1	RARS2	RPGRIP1	SCN5A	SIK1	SLC27A4
ODF2L	PCDH19	PGK1	PKP2	POMGNT2	PRKCG	RAB10	RASA1	RPGRIP1L	SCN8A	SIL1	SLC27A5
OFD1	PCDH7	PGM1	PLA2G4A	POMK	PRKCSH	RAB11B	RASAL1	RPH3A	SCN9A	SIN3A	SLC2A1
OGDH	PCDHB4	PGM3	PLA2G4B	POMT1	PRKD1	RAB12	RAX	RPIA	SCNN1A	SIRPA	SLC2A10
OGT	PCGF2	PGRMC1	PLA2G4C	POMT2	PRKN	RAB14	RBBP8	RPL10	SCNN1B	SIX1	SLC2A13
OPA1	PCK1	PHACTR1	PLA2G4D	POP1	PRKRA	RAB15	RBCK1	RPS19	SCNN1G	SIX3	SLC2A2
OPA3	PCLO	PHC1	PLA2G4E	POR	PRMT7	RAB18	RBFOX1	RPS23	SCO1	SIX5	SLC2A3
OPCML	PCNT	PHF10	PLA2G4F	PORCN	PRMT9	RAB1A	RBM10	RPS6KA3	SCO2	SKI	SLC30A10
OPHN1	PCSK1	PHF21A	PLA2G6	POU1F1	PRNP	RAB21	RBM20	RRAS	SCP2	SKIV2L	SLC30A10
OPLAH	PCSK9	PHF6	PLAA	PPA2	PRODH	RAB23	RBM28	RRM2B	SCRIB	SLC12A3	SLC30A3
OPTN	PCYT1A	PHF8	PLCB1	PPCDC	PROP1	RAB24	RBM8A	RSPH1	SCYL1	SLC12A5	SLC30A9
OR5M1	PDCD10	PHGDH	PLCB2	PPCS	PROX2	RAB25	RBP4	RSPH3	SDCCAG8	SLC12A6	SLC31A1
ORC1	PDE10A	PHIP	PLCB3	PPFIA2	PRPS1	RAB26	RBPJ	RSPH4A	SDHA	SLC13A5	SLC32A1
ORC4	PDE2A	PHKA1	PLCB4	PPFIA4	PRRG1	RAB27A	RD3	RSPH9	SDHAF1	SLC16A1	SLC33A1
ORC6	PDE4D	PHKA2	PLCE1	PPM1B	PRRG3	RAB27B	RDH12	RSPO4	SDHAF2	SLC16A2	SLC35A1
OSGEP	PDE6D	PHKB	PLCL1	PPM1D	PRRT2	RAB2A	RECQL4	RTEL1	SDHAF3	SLC17A5	SLC35A2
OTC	PDE6G	PHKG1	PLCXD1	PPM1K	PRSS12	RAB2B	REEP1	RTL9	SDHAF4	SLC17A6	SLC35A3
OTOGL	PDGFB	PHKG2	PLD1	PPOX	PRSS56	RAB30	REEP2	RTN1	SDHB	SLC17A7	SLC35C1
OTUD6B	PDGFRB	PHOX2B	PLD2	PPP1CA	PRUNE1	RAB31	RELN	RTN2	SDHC	SLC17A8	SLC35D1
OTULIN	PDHA1	PHYH	PLD3	PPP1CB	PRX	RAB33A	RENBP	RTN3	SDHD	SLC18A1	SLC36A2
OTX2	PDHB	PHYKPL	PLEC	PPP1CC	PSAP	RAB33B	REPS1	RTN4	SEC22B	SLC18A2	SLC37A4
OXA1L	PDHX	PI4K2A	PLEKHG5	PPP1R15B	PSAT1	RAB35	RERE	RTN4IP1	SEC23B	SLC18A3	SLC38A1
OXCT1	PDK1	PI4KA	PLK4	PPP1R1B	PSEN1	RAB39A	RET	RTTN	SEC24D	SLC19A2	SLC38A2
P2RY4	PDK2	PIBF1	PLN	PPP2CA	PSEN2	RAB39B	RETREG1	RUBCN	SEC63	SLC19A3	SLC39A13
P2RY8	PDK3	PICALM	PLOD1	PPP2CB	PSMA7	RAB3A	RF2BPL	RUNX2	SELENOI	SLC1A2	SLC39A14
P3H1	PDK4	PIEZO2	PLOD2	PPP2R1A	PSMB8	RAB3b	RFT1	RUSC2	SEMA6A	SLC1A3	SLC39A4
P4HB	PDLIM3	PIGA	PLOD3	PPP2R1B	PSMD10	RAB3C	RFX6	RYR1	SEPSECS	SLC1A4	SLC39A8
PABPC5	PDP1	PIGC	PLP1	PPP2R2A	PSMD12	RAB3D	RGN	RYR2	SERAC1	SLC1A7	SLC3A1
PACS1	PDP2	PIGG	PLPB1	PPP2R2B	PSPH	RAB3GAP1	RGS7	RYR3	SERPINI1	SLC20A2	SLC40A1
PACS2	PDPR	PIGH	PLPBP	PPP2R2C	PTCD1	RAB3GAP2	RHEB	S100B	SET	SLC20A2	SLC41A1
PAFAH1B1	PDSS1	PIGL	PLXNB3	PPP2R2D	PTCD3	RAB40AL	RHOB	SACS	SETBP1	SLC22A4	SLC45A1
PAH	PDSS2	PIGM	PMM2	PPP2R3A	PTCH1	RAB4A	RHOBTB2	SALL1	SETD1A	SLC22A5	SLC46A1
PAK1	PDX1	PIGN	PMP22	PPP2R3B	PTCHD1	RAB4B	RIMS1	SALL4	SETD1B	SLC24A1	SLC4A1
PAK3	PDXK	PIGO	PMPCA	PPP2R3C	PTDSS1	RAB5A	RIOK3	SAMD9	SETD2	SLC24A2	SLC4A11
PALB2	PDYN	PIGQ	PMPCB	PPP2R5A	PTEN	RAB5B	RIPK2	SAMD9L	SETD3	SLC24A3	SLC4A4
PANK2	PECR	PIGT	PMS2	PPP2R5B	PTF1A	RAB5C	RIPK4	SAMHD1	SETD5	SLC24A4	SLC52A1
PAPSS2	PEPD	PIGV	PNKD	PPP2R5C	PTH1R	RAB6B	RIT1	SAR1B	SETDB2	SLC24A5	SLC52A2
PARK15	PER2	PIGW	PNKP	PPP2R5D	PTHLH	RAB7A	RLIM	SARDH	SETX	SLC24A6	SLC52A3
PARK2	PET100	PIGY	PNLIP	PPP2R5E	PTPN11	RAB8A	RMND1	SARS2	SF3B4	SLC25A1	SLC5A1
PARK7	PET117	PIK3C3	PNP	PPP3CA	PTPN21	RAB8B	RMRP	SATB2	SFI1	SLC25A12	SLC5A2
PARK9	PEX1	PIK3CA	PNPLA4	PPP3CB	PTPN23	RAB9B	RNASEH1	SBDS	SFXN4	SLC25A13	SLC5A5
PARKIN	PEX10	PIK3CB	PNPLA6	PPP3CC	PTPN9	RABL6	RNASEH2A	SBF1	SGC	SLC25A15	SLC5A6
PARN	PEX11A	PIK3CD	PNPLA8	PPT1	PTPRE	RAC1	RNASEH2B	SBF2	SGCA	SLC25A19	SLC5A7
PARP1	PEX11B	PIK3CG	PNPO	PQB1	PTPRN2	RAD21	RNASEH2C	SC5D	SGCD	SLC25A2	SLC63
PARS2	PEX12	PIK3R1	PNPT1	PQBP1	PTPRZ1	RAD50	RNASET2	SCAMP1	SGCE	SLC25A20	SLC6A1
PASD1	PEX13	PIK3R2	POC1A	PRDM12	PTRH2	RAD51	RNF113A	SCAMP3	SGPL1	SLC25A21	SLC6A17
PAX2	PEX14	PIK3R3	POC1B	PRDM8	PTS	RAD51C	RNF125	SCAMP5	SGSH	SLC25A22	SLC6A19
PAX3	PEX16	PIK3R5	POGLUT1	PRDX4	PUDP	RAF1	RNF135	SCAPER	SH3PXD2B	SLC25A24	SLC6A2
PAX6	PEX19	PIK3R6	POGZ	PREPL	PUF60	RAI1	RNF168	SCARB1	SH3TC2	SLC25A26	SLC6A20
PAX7	PEX2	PIN4	POLA1	PRF1	PUM1	RALA	RNF170	SCARB2	SHANK1	SLC25A3	SLC6A3
PAX8	PEX26	PINK1	POLD1	PRICKLE1	PURA	RALGDS	RNF216	SCARF2	SHANK2	SLC25A32	SLC6A3
PAX9	PEX3	PINK1	POLG	PRICKLE2	PUS1	RANBP17	RNU4ATAC	SCLT1	SHANK3	SLC25A38	SLC6A4
PBRM1	PEX5	PITRM1	POLG1	PRICKLE3	PYCR1	RANBP2	ROBO3	SCN10A	SHH	SLC25A4	SLC6A5
PBX1	PEX6	PITX2	POLG2	PRKACA	PYCR2	RAP2A	ROGDI	SCN11A	SHOC2	SLC25A40	SLC6A7
PC	PEX7	PITX3	POLH	PRKACB	PYGL	RAP2B	ROR2	SCN1A	SHOX	SLC25A42	SLC6A8

SLC6A9	*SOX3*	*STX1A*	*TAZ*	*TGM6*	*TOP2A*	*TSEN34*	*UFC1*	*VLDLR*	*XPC*		
SLC7A10	*SOX4*	*STX1B*	*TBC1D20*	*TH*	*TOP3A*	*TSEN54*	*UFM1*	*VPS11*	*XPNPEP3*		
SLC7A14	*SOX5*	*STX2*	*TBC1D23*	*THAP1*	*TOPORS*	*TSFM*	*UGT1A1*	*VPS13A*	*XPR1*		
SLC7A7	*SOX6*	*STX3*	*TBC1D24*	*THG1L*	*TOR1A*	*TSHB*	*UMOD*	*VPS13B*	*XRCC1*		
SLC7A9	*SOX9*	*STX6*	*TBC1D32*	*THOC2*	*TP63*	*TSHR*	*UMPS*	*VPS13C*	*XRCC4*		
SLC9A1	*SPAG1*	*STX7*	*TBC1D7*	*THOC6*	*TPH1*	*TSPAN7*	*UNC13A*	*VPS13D*	*XYLT1*		
SLC9A6	*SPART*	*STXBP1*	*TBC1D8B*	*THRA*	*TPH2*	*TSPAN8*	*UNC13B*	*VPS16*	*XYLT2*		
SLC9A9	*SPAST*	*STXBP2*	*TBCD*	*THRB*	*TPK1*	*TTBK2*	*UNC13C*	*VPS33B*	*YAP1*		
SLCO1B1	*SPATA5*	*STXBP5*	*TBCE*	*THUMPD1*	*TPM1*	*TTC19*	*UNC13D*	*VPS35*	*YARS*		
SLCO1B3	*SPATA7*	*STXBP6*	*TBCK*	*THY1*	*TPMT*	*TTC1B*	*UNC80*	*VPS37A*	*YARS2*		
SLCSA6	*SPECC1L*	*SUCLA2*	*TBK1*	*TIMM22*	*TPP1*	*TTC21B*	*UPB1*	*VPS45A*	*YEATS2*		
SLX4	*SPEG*	*SUCLG1*	*TBL1XR1*	*TIMM44*	*TPPP*	*TTC37*	*UPF3B*	*VPS53*	*YME1L1*		
SMAD3	*SPG11*	*SUCLG2*	*TBP*	*TIMM50*	*TPRG1L*	*TTC7A*	*UQCC1*	*VRK1*	*YWHAG*		
SMAD4	*SPG11*	*SUFU*	*TBR1*	*TIMM8A*	*TRAF3IP1*	*TTC8*	*UQCC2*	*VSX2*	*YWHAQ*		
SMARCA1	*SPG20*	*SUGCT*	*TBX1*	*TIMMDC1*	*TRAF7*	*TTI2*	*UQCC3*	*VTI1A*	*YWHAZ*		
SMARCA2	*SPG21*	*SUMF1*	*TBX15*	*TINF2*	*TRAK1*	*TTN*	*UQCR10*	*WAC*	*YY1*		
SMARCA4	*SPG7*	*SUOX*	*TBX20*	*TITF*	*TRAK2*	*TTPA*	*UQCR11*	*WARS2*	*ZBTB16*		
SMARCAL1	*SPR*	*SURF1*	*TBX22*	*TK2*	*TRAP1*	*TTR*	*UQCRB*	*WASF1*	*ZBTB18*		
SMARCB1	*SPRED1*	*SV2A*	*TBX3*	*TKTL1*	*TRAPPC1*	*TUB8A*	*UQCRC1*	*WASHC4*	*ZBTB20*		
SMARCC1	*SPRTN*	*SV2B*	*TBX4*	*TLK2*	*TRAPPC11*	*TUBA1A*	*UQCRC2*	*WASHC5*	*ZBTB24*		
SMARCC2	*SPRY3*	*SV2C*	*TBX5*	*TLR8*	*TRAPPC12*	*TUBA1B*	*UQCRFS1*	*WDFY1*	*ZBTB40*		
SMARCD1	*SPTAN1*	*SVBP*	*TBXAS1*	*TM4SF20*	*TRAPPC2*	*TUBA3E*	*UQCRH*	*WDFY3*	*ZC3H14*		
SMARCD2	*SPTBN2*	*SVOP*	*TCAP*	*TM6SF2*	*TRAPPC3*	*TUBA4A*	*UQCRQ*	*WDPCP*	*ZC3H7A*		
SMARCD3	*SPTLC1*	*SYN1*	*TCEAL3*	*TMCO1*	*TRAPPC5*	*TUBA8*	*UROC1*	*WDR11*	*ZC4H2*		
SMARCE1	*SPTLC2*	*SYN2*	*TCF12*	*TMED10*	*TRAPPC6A*	*TUBAL3*	*UROD*	*WDR13*	*ZCCHC12*		
SMC1A	*SQSTM1*	*SYN3*	*TCF20*	*TMEM106B*	*TRAPPC6B*	*TUBB*	*UROS*	*WDR19*	*ZCCHC8*		
SMC3	*SRC*	*SYNCRIP*	*TCF4*	*TMEM107*	*TRAPPC8*	*TUBB2A*	*USB1*	*WDR26*	*ZDHHC15*		
SMCHD1	*SRCAP*	*SYNE1*	*TCIRG1*	*TMEM126A*	*TRAPPC9*	*TUBB2B*	*USF1*	*WDR34*	*ZDHHC9*		
SMN1	*SRCIN1*	*SYNGAP1*	*TCN1*	*TMEM126B*	*TREH*	*TUBB3*	*USH1C*	*WDR35*	*ZEB2*		
SMO	*SRD5A3*	*SYNGR1*	*TCN2*	*TMEM132E*	*TREM2*	*TUBB4A*	*USH1G*	*WDR4*	*ZFHX4*		
SMOC1	*SREBF2*	*SYNGR3*	*TCOF1*	*TMEM135*	*TREX1*	*TUBB6*	*USH2A*	*WDR45*	*ZFP57*		
SMPD1	*SRGAP3*	*SYNJ1*	*TCP10L2*	*TMEM138*	*TREX2*	*TUBG1*	*USP18*	*WDR45B*	*ZFX*		
SMPD4	*SRPX2*	*SYNJ1*	*TCTEX1D2*	*TMEM163*	*TRHR*	*TUBGCP4*	*USP27X*	*WDR48*	*ZFYVE26*		
SMS	*SRRT*	*SYNPR*	*TCTN1*	*TMEM165*	*TRIM2*	*TUBGCP6*	*USP7*	*WDR60*	*ZFYVE27*		
SNAP23	*SRY*	*SYP*	*TCTN2*	*TMEM199*	*TRIM32*	*TUFM*	*USP8*	*WDR62*	*ZIC1*		
SNAP25	*SSBP1*	*SYPL1*	*TCTN3*	*TMEM216*	*TRIM37*	*TULP1*	*USP9X*	*WDR63*	*ZIC2*		
SNAP29	*SSR4*	*SYT1*	*TDO2*	*TMEM231*	*TRIM8*	*TUSC3*	*UTP14A*	*WDR73*	*ZIC3*		
SNAP47	*ST3GAL3*	*SYT12*	*TDP1*	*TMEM237*	*TRIO*	*TWIST1*	*UTRN*	*WDR81*	*ZMPSTE24*		
SNAPIN	*ST3GAL5*	*SYT14*	*TDP2*	*TMEM240*	*TRIP11*	*TWIST2*	*UVSSA*	*WFS1*	*ZMYM3*		
SNCA	*ST5*	*SYT17*	*TECPR2*	*TMEM260*	*TRIP12*	*TWNK*	*VAC14*	*WHRN*	*ZMYM6*		
SNCAIP	*STAB2*	*SYT2*	*TECR*	*TMEM43*	*TRIP13*	*TXN2*	*VAMP1*	*WNK1*	*ZMYND10*		
SNCB	*STAG1*	*SYT5*	*TEK*	*TMEM5*	*TRIP4*	*TXNDC15*	*VAMP2*	*WNK3*	*ZMYND11*		
SNIP1	*STAG2*	*SYTL4*	*TELO2*	*TMEM65*	*TRIT1*	*TXNL4A*	*VAMP3*	*WNT1*	*ZMYND12*		
SNORD118	*STAMBP*	*SYTL5*	*TENM1*	*TMEM67*	*TRMT1*	*TXNRD1*	*VAMP4*	*WNT10B*	*ZNF148*		
SNRPB	*STAR*	*SZT2*	*TEPSIN*	*TMEM70*	*TRMT10A*	*TYMP*	*VAMP7*	*WNT3*	*ZNF292*		
SNRPN	*STARD8*	*TAB2*	*TERT*	*TMLHE*	*TRMT10C*	*TYR*	*VAPA*	*WNT4*	*ZNF335*		
SNTB2	*STAT1*	*TACO1*	*TFAM*	*TMPRSS6*	*TRMT5*	*TYROBP*	*VAPB*	*WNT5A*	*ZNF41*		
SNTG1	*STAT2*	*TAF1*	*TFAP2A*	*TMTC3*	*TRMU*	*TYRP1*	*VARS*	*WNT7A*	*ZNF423*		
SNX14	*STAT5B*	*TAF13*	*TFAP2B*	*TMX2*	*TRNT1*	*UBA1*	*VARS2*	*WRAP53*	*ZNF425*		
SNX3	*STIL*	*TAF2*	*TFB2M*	*TMX4*	*TRPA1*	*UBA5*	*VAT1*	*WRN*	*ZNF526*		
SNX5	*STOML2*	*TAF6*	*TFE3*	*TNIK*	*TRPC1*	*UBE2A*	*VCL*	*WT1*	*ZNF592*		
SOBP	*STRA6*	*TAF7L*	*TFG*	*TNK2*	*TRPM1*	*UBE3A*	*VCP*	*WWC3*	*ZNF599*		
SOD1	*STRADA*	*TALDO1*	*TFR2*	*TNKS2*	*TRPM6*	*UBE3B*	*VDAC1*	*WWOX*	*ZNF674*		
SON	*STS*	*TANC2*	*TGDS*	*TNNC1*	*TRPS1*	*UBQLN1*	*VDAC2*	*XDH*	*ZNF711*		
SOS1	*STT3A*	*TANGO2*	*TGFB1*	*TNNI3*	*TRPV4*	*UBQLN2*	*VDR*	*XDP*	*ZNF713*		
SOS2	*STT3B*	*TAOK1*	*TGFB2*	*TNNT2*	*TSC1*	*UBR1*	*VEGFA*	*XIAP*	*ZNF81*		
SOX10	*STUB1*	*TAPT1*	*TGFB3*	*TNPO2*	*TSC2*	*UBR4*	*VHL*	*XIST*	*ZSWIM6*		
SOX11	*STX11*	*TARDBP*	*TGFBR1*	*TNRC6B*	*TSC22D3*	*UBR7*	*VIP*	*XK*			
SOX17	*STX12*	*TARS2*	*TGFBR2*	*TOE1*	*TSEN15*	*UBTF*	*VIPAS39*	*XKRX*			
SOX2	*STX16*	*TAT*	*TGIF1*	*TONSL*	*TSEN2*	*UCHL1*	*VKORC1*	*XPA*			

Table S2: Neurotransmitter analysis of patient CSF, showing normal levels of neurotransmitters but a slight increase in HVA:5-HIAA levels.

CSF metabolite (age related reference ranges[1])	18 months	24 months
5-HIAA (89-367nmol/l)	138	182
HVA (154-867nmol/l)	603	499
HVA:5-HIAA (1.0-3.7)	**4.4** ↑	2.7
Dihydrobiopterin (0.4-13.9 nmol/l)	5.7	8.9
Tetrahydrobiopterin (8-57 nmol/l)	33	22
Total neopterin (7-65 nmol/l)	10	17
Pyridoxine (11-64 nmol/l)	38	29
5-MTHF (<2 years: 72-305 nmol/l; 2-5 years: 52-178nmol/l)	74	86

Table S3: Candidate homozygous variants identified in proband

Gene Transcript	Variant	Frequency (homozygote count)	CADD	PolyPhen2 (HumVar)	SIFT	Mutation Taster	Function	Expression	Disease phenotype (inheritance)	Notes
ATXN3 NM_004993.6	c.915_916ins17 p.G306Qfs*31	Absent	19.2	n/a	n/a	Benign (82|118)	Deubiquitinating enzyme	Ubiquitous	Machado-Joseph disease, MIM109150 (AD)	Adult-onset cerebellar degeneration; triplet repeat disease
CIC NM_001386298.1	c.4394C>A p.T1465N	0.048% (1)	21.9	Benign (0.021)	Tolerated (0.18)	Benign (14|86)	Transcriptional repressor	Ubiquitous	Intellectual developmental disorder, MIM617600 (AD)	Only truncating variants reported as pathogenic
DRD1 NM_000794.4	c.110C>A p.T37K	Absent	27.5	Probably damaging (0.99)	Deleterious (0.02)	Deleterious (89|11)	Dopamine receptor	Brain, especially basal ganglia	Nil	See text
GPR4 NM_005282.3	c.864_871 delinsACGT p.V289Rfs*49	Absent	n/a	n/a	n/a	Deleterious (168|32)	Proton-sensing GPCR; role in breathing control	Highest in adipose, kidney, lung, thyroid; low in brain	Nil	Close to 3' terminus
MUC16 NM_024690.2	C19511C>A p.S6504Y	0.39% (3)	15.64	Unavailable	n/a	Benign (0|100)	Mucus component	Mucosal membranes	Nil	.
ZAN NM_003386.3	c.5768del p.C1923Ffs*18	Absent	33	n/a	n/a	Deleterious (165|35)	Allows sperm to bind zona pellucida	Testis only	Nil	Expressed exclusively in sperm
ZFN354C NM_014594.2	c.55G>A p.V19M	0.0011% (0)	23.5	Probably damaging (0.968)	Deleterious (0)	Benign (8|92)	Transcriptional regulator: involved in vascular and bone development	Ubiquitous	Nil	.

Supplementary Figure Legends

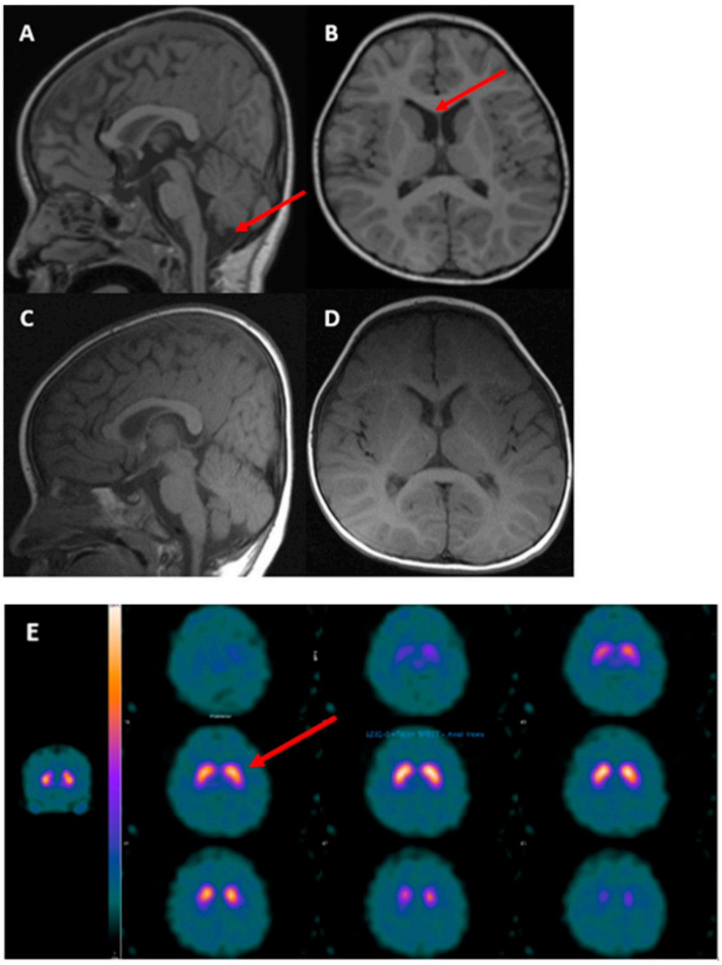

Figure S1: MRI investigations of proband (A) T1-weighted MRI. Top (left): midline sagittal, aged 36 months. (right): axial, aged, 36 months, showing minimally increased prominence of ventricular and extraventricularCSF spaces (red arrows). Bottom (left): midline sagittal, aged 13 months. (right): axial, aged 13 months. (B): DaTscan images, aged 35 months, showing normal tracer uptake indicated by normal outlining of the striatum (red arrow).

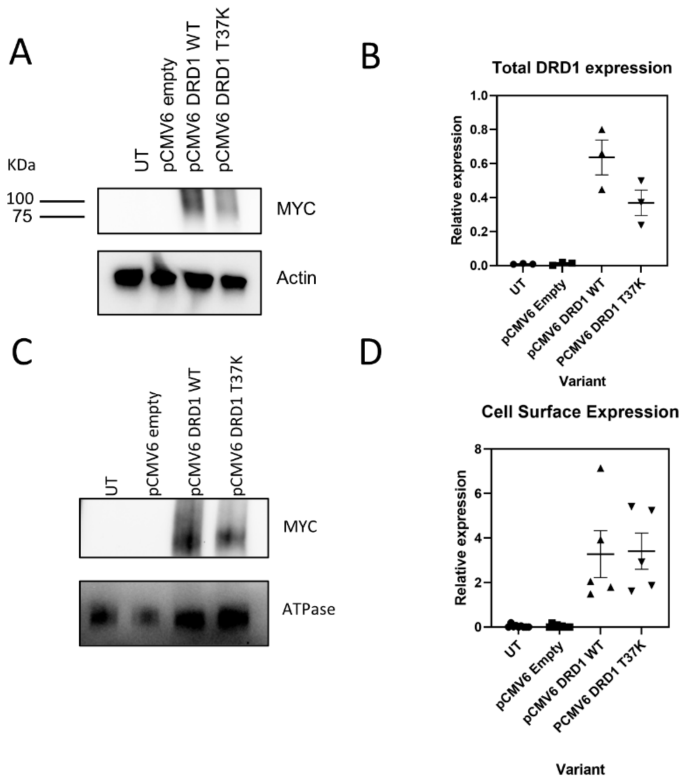

Figure S2: Expression and cell surface localisation of *DRD1*-T37K.

DRD1-WT and *DRD1*-T37K were overexpressed in HEK-293T cells. (A) Total protein expression was assessed using Western blotting, showing an ~80KDa band corresponding to Myc-tagged D_1 protein. (B) Average Myc expression relative to β-actin loading control. Data is presented as mean +/- SEM (*t* test, n=9, p>0.05). (C) Cell surface localisation investigated using biotinylation and immunoblotting analysis. (D) Average Myc expression in cells relative to Na^+/K^+ ATPase (cell surface marker). Data is presented as mean +/- SEM (*t* test, n=9, p>0.05)

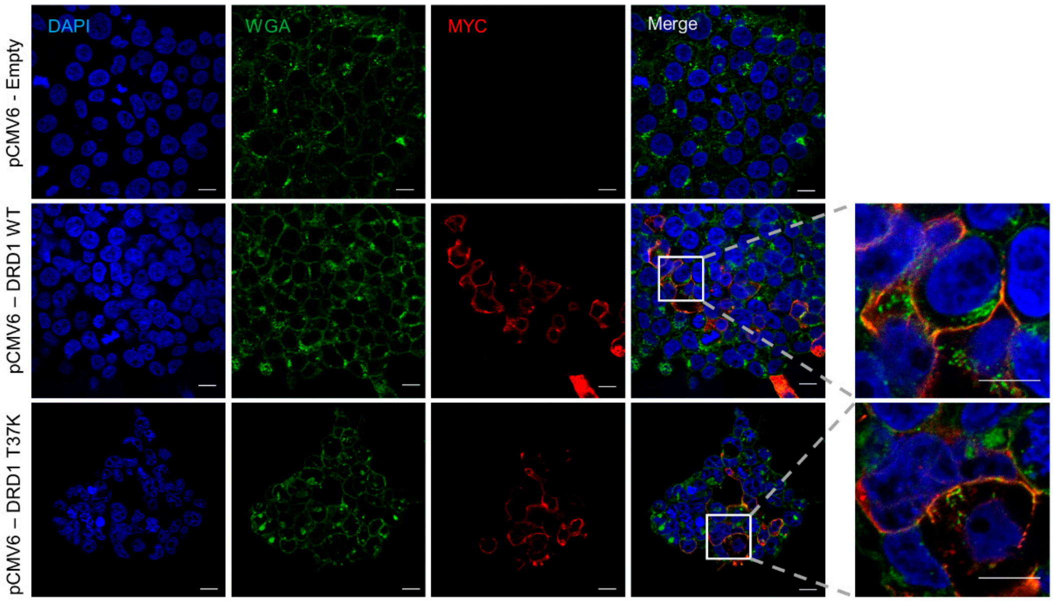

Figure S3: Cell surface localisation of DRD1 by immunofluorescence

HEK-293T cells transfected with either *DRD1*-WT or *DRD1*-T37K were fixed and stained with anitbodies against MYC. Representative images show MYC co-localisation with WGA, a cell surface marker. Scale bars indicate 10 nm

Video S1: Patient video: Oculogyric crises and exacerbations of dystonia displayed by the pro-band. Clip 1: the proband is approximately 18 months old. Note dystonic posturing of all limbs; opisthotonos and distress. Clips 2a and 2b: the proband is approximately three years old. In addition to the previous signs, striatal toe, dystonic jaw opening and (in 2b) fixed bilateral upwards eye deviation is seen. Clip 3: the proband is four years old, with similar movement disorder semiology.

References

1. Hyland K, Surtees RAH, Heales SJR, Bowron A, Howells DW, Smith I. Cerebrospinal fluid concentrations of pterins and metabolites of serotonin and dopamine in a pediatric reference population. *Pediatr Res*. 1993;34(1):10-14. doi:10.1203/00006450-199307000-00003
